# Comparative Investigation of Collagen-Based Hybrid 3D Structures for Potential Biomedical Applications

**DOI:** 10.3390/ma14123313

**Published:** 2021-06-15

**Authors:** Geta David, Alexandra I. Bargan, Mioara Drobota, Adrian Bele, Irina Rosca

**Affiliations:** 1 Department of Natural and Synthetic Polymers, “Gh. Asachi” Technical University of Iasi, 71A Bd. D. Mangeron, 700050 Iasi, Romania; 2 “Petru Poni” Institute of Macromolecular Chemistry of Romanian Academy, 41A Gr. Ghica Voda Alley, 700487 Iasi, Romania; miamiara@icmpp.ro (M.D.); bele.adrian@icmpp.ro (A.B.); rosca.irina@icmpp.ro (I.R.)

**Keywords:** hybrid hydrogels, biopolymers, poly(ε-caprolactone), drug delivery, dressings

## Abstract

Collagen is a key component for devices envisaging biomedical applications; however, current increasing requirements impose the use of multicomponent materials. Here, a series of hybrid collagen-based 3D materials, comprising also poly(ε-caprolactone) (PCL) and different concentrations of hyaluronic acid (HA)—in dense, porous or macroporous form—were characterized in comparison with a commercially available collagen sponge, used as control. Properties, such as water uptake ability, water vapour sorption, drug loading and delivery, were investigated in correlation with the material structural characteristics (composition and morphology). Methylene blue (MB) and curcumin (CU) were used as model drugs. For spongeous matrices, it was evidenced that, in contrast to the control sample, the multicomponent materials favor improved sustained release, the kinetics being controlled by composition and cross-linking degree. The other characteristics were within an acceptable range for the intended purpose of use. The obtained results demonstrate that such materials are promising for future biomedical applications (wound dressings and lab models).

## 1. Introduction

Collagen-based 3D materials are extensively used in biomedical applications (scaffolds in tissue engineering, lab models for drug screening, modern wound dressings, and drug delivery devices) due to their protein properties. The most envisaged are: biocompatibility and inherent bio-functionality enabling cells’ proliferation and differentiation, coupled with haemostatic properties, low immunogenicity, a rich chemistry and high water-holding capacity [1,2]. A porous architecture facilitates cell adhesion and improves flexibility and substance permeability, finally promoting 3D structure biomimicry [3,4]. However, the application of this protein for biomedical devices, as a unique raw source, is limited by some disadvantages, such as the poor mechanical and antibacterial properties, fast degradation, modification and processing difficulties, some batch-to-batch variations and risk of human transgenic disease transmission [1]. Cross-linking (by physical, chemical or combined techniques) and combination with other polymers (natural or synthetic) or materials (i.e., ceramics) may be used as topical, convenient routes to avoid the biopolymer drawbacks and to improve/control the physico-chemical and biological properties of collagen-based materials, to meet specific needs [1,5,6,7].

Nowadays collagen-based materials in different forms (sponge, films, micro-nanoparticles, injectable formulations, etc.) are still intensively studied, envisaging biomedical uses, and some commercialized devices are already clinically applied. Between them, hydrogels are known to meet most of the desirable characteristics for the design of an ideal dressing [8].

More components may simultaneously address different aspects of wound healing [7,8,9]. Thus, modern dressings contain often polymers with wound-healing activity (biological dressings) and include pharmacological agents, being used as local drug delivery systems, in order to improve effectiveness and to ensure a sustained action, reducing also the risk of systemic toxicity [8,10,11]. Such dressings (multicomponent or composite-engineered constructs) are actively involved in all the stages of wound healing. Very good results were reported for the collagen–glycosaminoglycan (CG) system, such as the most known, commercial bilayer dermal substitute Integra (porous matrix containing collagen and glycosaminoglycan, covered with a semi-permeable polysiloxane layer) [12]. Spongy sheet based on collagen and hyaluronic acid containing epidermal growth factor and vitamin C derivative was found to more effectively promote granulation tissue formation associated with angiogenesis, as compared with other wound dressings [13]. Recent work developed in an effort to obtain improved, injectable CG formulation (without other bioactives), making use of enzymatic cross-linking, pointed out its quick healing properties, which are attributed to the role of the hydrogel in promoting the spontaneous formation of new vasculature, epithelial layer and collagen. This gave rise to an increased rate of healing for the CS system (collagen-hyaluronic acid), as compared to commercial drug or individual hydrogels of the components [14]. It is worth noting that in situ forming hydrogels (sprayable or injectable) for application as wound dressings constitute a major challenge in current research. With respect to the increasing demand for higher performance dressings, there is a continuous interest for the development of novel hydrogel-based dressings, with multifunctional properties (e.g., antibacterial ability, biodegradability, multi-responsiveness, and injectability), improved stability and performances, and low production costs/simple preparative protocol [7,15,16]. Multicomponent, hybrid and composite hydrogels are considered to be a solution.

In this context, aiming to develop new modern wound dressings or lab models, this study is dedicated to a comparative evaluation of hybrid 3D structures, containing natural and synthetic polymers (i.e., atelocollagen, hyaluronic acid and poly(ε-caprolactone) derivatives), with different architectures (dense film, porous or macroporous sheet) and compositions. Their characteristics (morphology, swelling behaviour, water vapour sorption, drug loading/delivery ability, and anti-microbial activity of drug loaded system) were assessed by comparison with a marketable collagen wound dressing (Suprasorb^®^C- Lohmann & Rauscher, Rengsdorf, Germany). Thus, the usual requirements, targeting aspects of different phases in the wound healing process, were considered, i.e., moist wound environment, oxygen and water vapour circulation and bacterial protection.

The swelling, cross-linking density, degradation rate and delivery kinetics can be tuned according the application by selecting the composition and the preparative method. Here, while joined collagen and hyaluronic acid are known to accelerate wound healing [12,17], the poly(ε-caprolactone) derivative, used as a cross-linker, controls the mechanical stability and degradability rate of the studied constructs [18] and may modify the release kinetics of included therapeutics. Moreover, hyaluronan interacts directly with cell surface receptors; thus, it is able to recognize overexpressed specific receptors on the tumour cells’ surface, and has an important role in tumour growth and metastasis, aspects that recommend its use in the design of both controlled drug delivery systems and lab models (to obtain insights on carcinogenesis) [19]. Dimethylsilandiol hyaluronate, used here, is known to have also cyto-stimulating properties [20]. Thus, such complex macromolecular materials could offer the possibility to be tuned to accommodate to the envisaged use requirements.

To evaluate loading/delivery behaviour for the investigated matrices, therapeutics from different classes were chosen as drug models, i.e., methylene blue (water-soluble heterocyclic aromatic compound), and curcumin (natural lipophilic polyphenol substance). Methylene blue is an old therapeutic dye of renewed interest with uses in antimicrobial chemotherapy, diagnostics, and different illness therapy (i.e., methemoglobinemia, encephalopathy, ischemia, vascular dementia and Alzheimer’s disease) [21]. Curcumin is a nutraceutical known for its anti-inflammatory, anti-oxidant, anti-tumour and wound-healing effects, and anti-infectious and neuroprotective activities [22]. The main drawbacks are its extremely low aqueous solubility and short half-life, involving limited bioavailability [23]. Even if it was demonstrated that its degradation products also have therapeutic effects, the development and improvement of strategies to address curcumin challenge are still of high interest [24].

## 2. Materials and Methods

### 2.1. Materials

Aqueous acidic solutions (pH ~ 2, HCl) of type I atelocollagen (AteCol) and poly(ε-caprolactone) diisocyanate (PCL-DI, Mn ~2.5 kDa) were prepared according to the protocols outlined in the literature [25,26]. Dimethylsilanediol hyaluronate (DMSHA) (aqueous solution of pH ~ 5.5, 0.6% with 0.3% hyaluronan of nonanimal origin with Mw of 1.8–2.2 MDa) was kindly donated by EXSYMOL S.A.M. (Monaco). Suprasorb C collagen wound dressings (Lohmann & Rauscher, Rengsdorf, Germany) were used as reference samples (Col LR). Curcumin (CU), ≥90% (reagent from Carl Roth, Karlsruhe, Germany) was used as received. Methylene blue (MB), phosphate buffered saline tablets (PBS, pH 7.4) and solvents (acetone p.a., dimethylsulfoxide puriss p.a.-DMSO, absolute ethanol for HPLC, glycerol ≥99.5%), purchased from Sigma–Aldrich (Darmstadt, Germany), were used without further purification. Triton X-100, poly(vinyl pyrrolidone) K15 were supplied by Sigma-Aldrich (Darmstadt, Germany). Bidistilled water or Milli Q ultrapure water was used in all experiments. The antibacterial activity of the investigated collagen-based materials loaded with curcumin was evaluated on the Gram-positive strain of *Staphylococcus aureus* ATTC 25923.

### 2.2. Hybrid 3D Structures Preparation

**Scheme 1:**
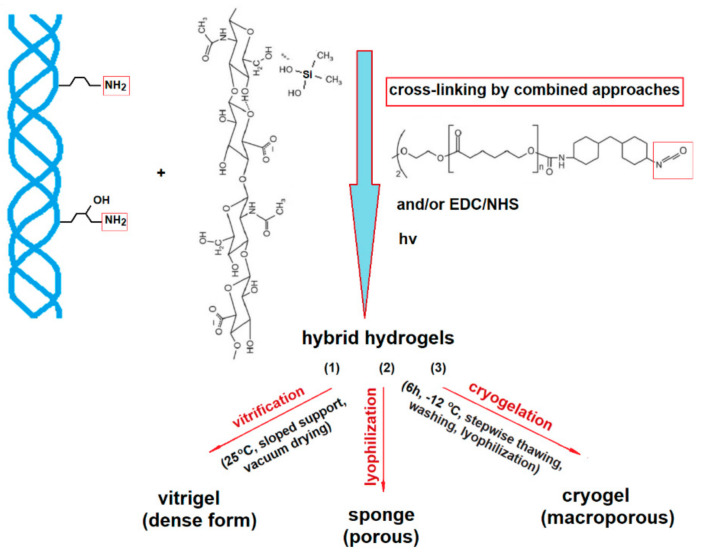
Schematic representation of the preparative protocol for the investigated hybrid 3D constructs.

The hybrid matrices were obtained according to previous published procedures, i.e., by: (1) vitrigel preparation strategy (implying a vitrification step) [27], (2) simple lyophilization [26] or (3) cryogelation followed by lyophilization [28] (Scheme 1).

Briefly, the appropriate amount of DMSHA solution was slowly, dropwise mixed under continuous stirring with the AteCol dispersion of a pH adjusted to ≥6.5 (with 1 M NaOH), and then PCL-DI cross-linker (as a solution in 2/3 *v*/*v* acetone/DMSO mixture containing Triton X-100 stabilizer) was added, according to the envisaged formulation. The resulted mixture was further homogenized through 5 min sonication and vacuum degassed. When cryogelation protocol was used, the solid concentration was brought to 0.9 wt%. The resulting multicomponent dispersions were then processed in accordance with the selected procedure; specifically, they were: (1) cast on a sloped support disposed in a desiccator, and slowly vacuum dried to form a dense film [27]; (2) frozen at −20 °C, followed by lyophilization (Alpha 1–4 LSC type CHRIST freeze dryer-Martin Christ, Osterode, Germany) [26] or (3) frozen according the cryogelation stepwise selected regime [28], and subsequently lyophilized. In all cases, the samples were subjected to UV irradiation (Osram HBO 200 W super pressure mercury lamp-Osram, Munich, Germany), before (in frozen state- procedure 3) or after drying/freeze-drying (protocols 1 and 2), to complete chemical long-range cross-linking with physical short-range cross-linking. The adopted code was CH_x_P_y_-z,n, where C, H, P are the abbreviations for the components AteCol, DMSHA, and PCL, while x represents wt% DMSHA (relative to collagen), y is wt% PCL-DI (relative to biopolymers), z indicates the irradiation duration (min), and n the adopted procedure. For comparison, samples of lyophilized AteCol sponge were crosslinked by using the EDC/NHS system in ethanol/water mixture, eventually followed by a new cross-linking with PCL-DI (disposed in acetone-DMSO), namely C_EN_,2 and C_EN_P_2_,2 [26]. One of the double cross-linked samples was also subjected to UV irradiation (C_EN_P_2_-30,2).

### 2.3. Methods

The infrared analysis of the samples before and after drug model loading was performed with a Bruker Vertex 70 spectrometer-Ettlingen, Germany, equipped with a diamond crystal and a single reflexion at incidence angle of 45°, using the Attenuated Total Reflection FT-IR mode (ATR-FTIR) in the 4000–600 cm^−1^ region. All spectra were recorded at room temperature with a resolution of 2 cm^−1^, with 64 scans performed.

The UV-VIS spectroscopic determinations were realized on a UV 6300-PC (VWR) spectrophotometer-Europe VWR, International Europe, Leuven, Belgium, at λ = 585 nm and/or 291 nm for methylene blue and 429 nm for curcumin. Standard calibration curves were used for drug content quantification.

The samples’ morphology was investigated with an Environmental Scanning Electron Microscope (ESEM) type Quanta 200 (FEI Company, Hillsboro, OR, USA), operating at 20 kV with a large field detector (LFD), in Low vacuum mode. The ESEM studies were performed on samples fixed on copper supports, directly (without sputter coating), using a working distance of 9–14 mm.

The water uptake was evaluated gravimetrically at room temperature by measuring the weight increase of the samples immersed in bidistilled water, after gentle removal of excess surface water with filter paper, at predetermined time intervals until equilibrium was reached. The equilibrium swelling ratio (ESR) was calculated considering the weight of the swollen sample at equilibrium (Ws_∞_) and its initial weight in the dried state (W_d_), according to Equation (1):

(1) $$\mathrm{ESR}\;\left({\mathrm{gxg}}^{-x}\right)=\frac{\left(W_{s_{\infty }}-W_{d}\right)}{W_{d}}$$

Each test was performed in triplicate.

The dynamic water vapour sorption behaviour was studied with a IGAsorp (Hiden Analytical, Warrington, UK) system at 25 ± 0.3 °C, in the range of relative humidity from 0 to 90%, by using samples (~3 mg) preliminarily dried in nitrogen flow (250 mL/min, 25 °C), in triplicate. The dynamic sorption capacity was calculated with Equation (2):

(2) $$\mathrm{DVS}\;\left(\%\right)=\frac{\left(W_{\mathrm{RH}=90}-W_{\mathrm{RH}=0}\right)}{W_{\mathrm{RH}=0}}\times 100$$

The porosity was determined by liquid displacement, using an appropriate non solvent as displacement liquid [29]. Briefly, a dried sample of weight Ws was immersed in a precise graduated cylinder with glass cap containing the non solvent, for 5 min, with repeated evacuation–repressurization cycles until no air bubbles could be observed, to insure the efficient pores penetration. The sample was then extracted and weighed immediately (total weight—Wt). The porosity was calculated with the Formula (3):

(3) $$P\left(\%\right)=\frac{\frac{W_{t}-W_{s}}{\rho_{\mathrm{NS}}}}{\frac{W_{s}}{\rho_{s}}+\frac{W_{t}-W_{s}}{\rho_{\mathrm{NS}}}}\times 100$$

where $\rho_{s}$ is the sample density (obtained by picnometry) and $\rho_{NS}$ is the non solvent density.

The antibacterial activity was assessed by disk diffusion bioassays [30]. The microorganism (*Staphylococcus aureus* ATTC 25923) was stored at −80 °C in 10% glycerol. The bacterial strain was refreshed in Mueller–Hinton broth (Merck, Darmstadt, Germany) at 36 ± 1 °C, and afterward, was inoculated on Plate Count Agar plates (Merck, Darmstadt, Germany) for purity checking. A microbial suspension was prepared with this culture in sterile saline solution to obtain turbidity optically comparable to that of the 0.5 McFarland standards (yielding a suspension containing approximately 1 × 10^8^ CFU mL^−1^). A volume of 0.5 mL of this inoculum was spread onto Mueller–Hinton Agar in Petri dishes and the biopolymer sheets (10 × 10 mm, 3–4 mm thickness, approximately 20 mg) were added after the medium surface was dry. To evaluate the antimicrobial properties, the inhibition of growth was measured under standard conditions after 24 h of incubation at 36 ± 1 °C. The diameter of the inhibition zone around the films was measured using the ImageJ version 1.52t software (Rasband, W.S., ImageJ, U.S. National Institutes of Health, Bethesda, MD, USA, https://imagej.nih.gov/ij/, accessed on 21 February 2020, 1997–2018).

### 2.4. Drug Loading and Release

Loading of drug models was performed by absorption. Hydrogel samples (~20 mg) were immersed in a ratio of 2.2 mg/mL in the MB aqueous solution (0.1 mg/mL, at 37 °C) or in a curcumin solution in 3/2 *v*/*v* ethanol/water mixture (2 mg/mL, at 30 °C), respectively. After 6 h, the loaded samples were separated and freeze-dried, and maintained in dark conditions. The supernatant was subjected to spectrophotometric analysis of the unabsorbed drug to quantify the amount of loaded drug, with reference to appropriate standard curves.

The in vitro delivery study was carried out in phosphate buffer 0.01 M with a pH of 7.4. Briefly, dried loaded hydrogel pieces of about 20 mg were placed in 9 mL PBS solution, and maintained at 37 °C with gentle periodical shaking. At predetermined time intervals, aliquots of 2 mL were extracted and replaced with equal volumes of fresh PBS solution. The released drugs were quantified spectrophotometrically. For curcumin content quantification, the supernatant samples were completed with ethanol to obtain 3/2 *v*/*v* ethanol/water solutions, and eventually diluted. Experiments were performed in triplicate.

## 3. Results and Discussion

### 3.1. Water Uptake Ability and Water Vapour Sorption Behaviour

Materials envisaged for use as wound dressing should meet certain requirements related to their main functions: (a) to provide a moist environment, (b) to remove the excess exudates, but prevent desiccation of the wound; (c) to control the bacterial load, protecting the wound site from secondary infections, and (d) to insure patient comfort (appropriate mechanical properties). For the new, modern dressings, it is also desirable to use non-antigenic, biocompatible materials, which are also able to stimulate the growth factors, sometimes in conjunction with antibacterial agents.

The hemo/biocompatibility of specimens with similar composition as those investigated here was proved by in vitro and in vivo tests presented in earlier studies [27,28].

The ability to absorb and retain wound fluid and the water vapour permeability, important in exudate management, is dependent on the material characteristics. The ESR values of the collagen-based hydrogels investigated here, together with their porosity data, are given in Table 1. As can be observed, the control of the porosity and topographic features can be adjusted through the preparative method and recipe choice (type and amount of included polymers), especially considering the role of the cross-linker of PCL-DI and of the co-cross-linker of hyaluronic acid derivative.

**Figure 1:**
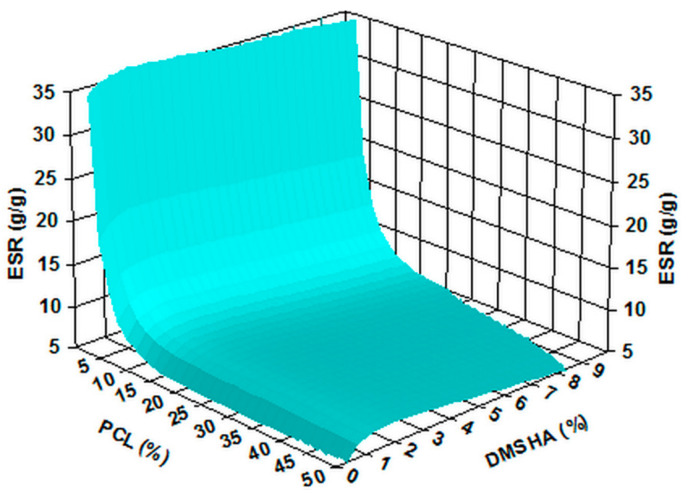
Three-dimensional representation of the investigated specimens’ swelling behaviour as a function of composition.

The 3D representation of the ESR as a function of composition, considering the swelling behaviour for the specimens with similar pore volume fraction and UV irradiation duration (of about 15 min), is shown in Figure 1. Most of the samples favour water absorption. As can be observed, modification by adding DMSHA/PCL results in a reduction in water sorption. There is a drastic decrease in PCL content, a hydrophobic, crystallisable biomaterial. Its presence strongly affects the 3D structure morphology and hydrophobic/hydrophilic balance, not only the cross-links’ density. Thus, the use of PCL to increase stability and mechanical properties must also take into consideration the effect on surface roughness and inner morphology, which affect the swelling behaviour. The hyaluronic acid derivative is a moisturizer component, but also has the ability to develop strong electrostatic interactions coupled with hydrogen bonding with proteins (in this case, collagen) [1,31]. This results in an earlier increase followed by a slight decrease in water absorption after about 4–5 wt% DMSHA, when it acts mainly as a co-cross-linker, improving material stability and mechanical resistance. Thus, an optimum balance must be selected between these characteristics, which is attained by means of composition control.

**Figure 2:**
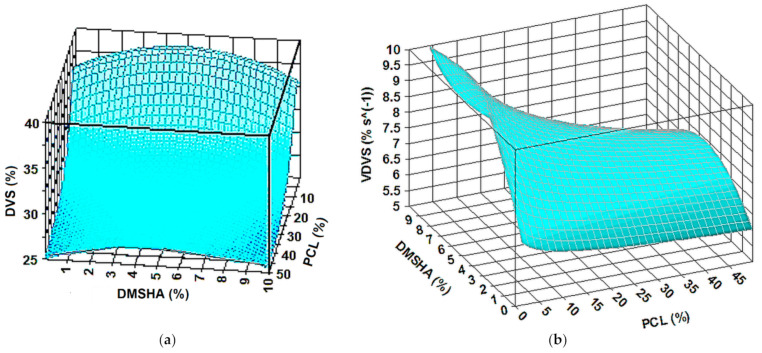
Water vapour sorption dependence on composition: (**a**) dynamic water vapour sorption capacity; (**b**) rate of dynamic water vapour sorption.

Similar behaviour can be observed for water vapour sorption (Figure 2), where the dynamic sorption capacity and water vapour sorption rates reach a maximum in the same range of values, i.e., 4–5% DMSHA (2–2.5% glycosaminoglycan relative to protein). This observation is in accordance with data reported in the literature [12] on collagen/glycosaminoglycan-based materials’ efficiency for skin regeneration (recommended for scaffolds and wound dressings).

**Figure 3:**
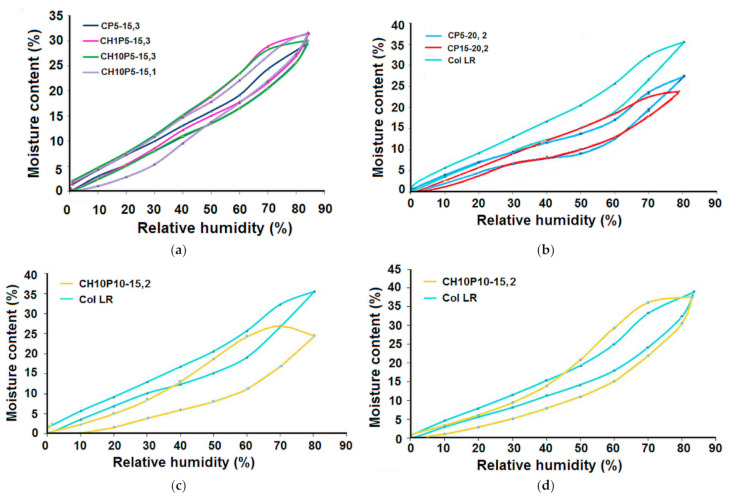
Typical water vapour sorption–desorption isotherms: effect of composition. Registration conditions: (**a**,**b**) and (**c**) equilibrium time 10/20 min; (**d**) equilibrium time 40/60 min.

Typical water vapour sorption–desorption isotherms for the investigated samples are shown in Figure 3, in comparison with the corresponding isotherms of the control sample (Col LR). The characteristic features are the sigmoidal shape and the presence of hysteresis, specific to the type IV isotherms, according to the IUPAC classification. They are reflecting adsorption via mono-multilayer (mainly multilayer) and capillary condensation in the pores.

The swelling and water vapour sorption behaviour strongly depend on material structure (composition, water affinity related to the presence of polar groups), surface area, and morphology (topography and porosity, pores’ sizes and shapes). Considering the obtained data, the pores’ sizes and shapes, as well as the presence of polar groups with water affinity, are more important than the pore volume. Thus, there are no significant differences between porous, macroporous and dense 3D structures (Figure 3a). Monolayer adsorption is more important in dense films (Figure 3a: sample CH_10_P_5_-15,1 comparative to CH_10_P_5_-15,3). The hysteresis loops and water vapour uptake levels are strongly influenced by hydrophobic/hydrophilic balance modification. This is evident for CH_10_P_5_-15,3 and CH_1_P_5_-15,3, with increased loops comparative to CP_5_-15,3, due to hydrophilic glycosaminoglycan (GAG) incorporation (Figure 3a). These observations are also highly sustained by the comparison of hysteresis for the control sample and CP_5_-20,2 or CP_15_-20,2 (increased hydrophobic/hydrophilic balance, similar porosity—Figure 3b). The importance of topography is evidenced in Figure 3c, by comparing the control sample and CH_10_P_10_-15,2, characterised by lower porosity, smaller pores, but increased content of GAG (with high water absorbent ability) and a cauliflower-type topography (due to GAG-protein complexation), creating a high surface for GAG exposing. Thus, the intriguing coupling of lower water absorbency with an increased water vapour sorption for the sample CH_10_P_10_-15,2 is related to its composition and to the specific topography. Moreover, this sample required an increased equilibrium time (40/60 min) comparative to the others (10/20 min) for correct registration (Figure 3c,d). The equilibrium is becoming more dificult to reach, especially for higher humidities, due also to possible cleavage of the physical bonds in the sample. The differences between the values of the weight gained by the sample CH_10_P_10_-15 when the established equilibrium time of the sorption/desoption isotherm is 10/20 min and when the equilibrium time of the isotherm is equal to 40/60 min are notable, almost 13.31%. For the reference sample, this difference is about 2.29%, meaning that the equilibrium sorption/desorption for every step is reached more easily.

**Figure 4:**
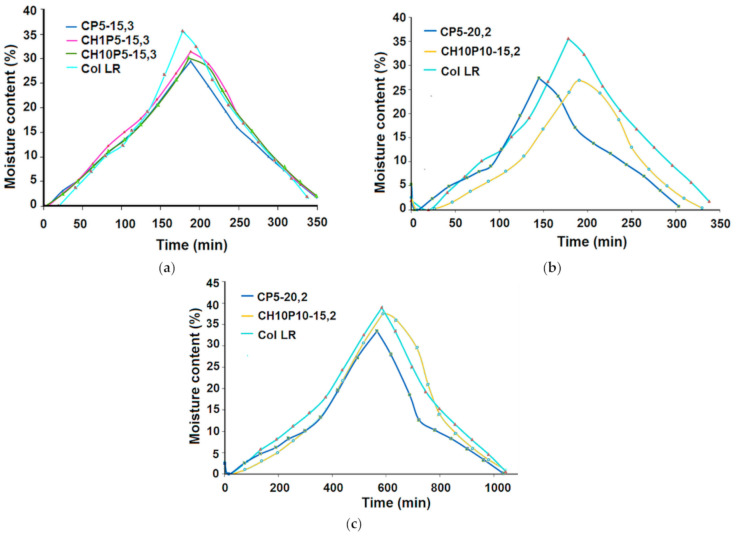
Kinetics of water sorption/desorption for different experimental conditions (equilibrium time): (**a**,**b**) equilibrium time 10/20 min; (**c**) equilibrium time 40/60 min.

Considering the kinetical plots (Figure 4), samples CH_1_P_5_-15,3 CH_10_P_5_-15,3 and CH_10_P_10_-15,2 are the most appropriate for efficiently removing any excess wound exudate.

### 3.2. Characterization of Drug Loaded Collagen-Based Matrices

#### 3.2.1. Loading with Drug Models

**Figure 5:**
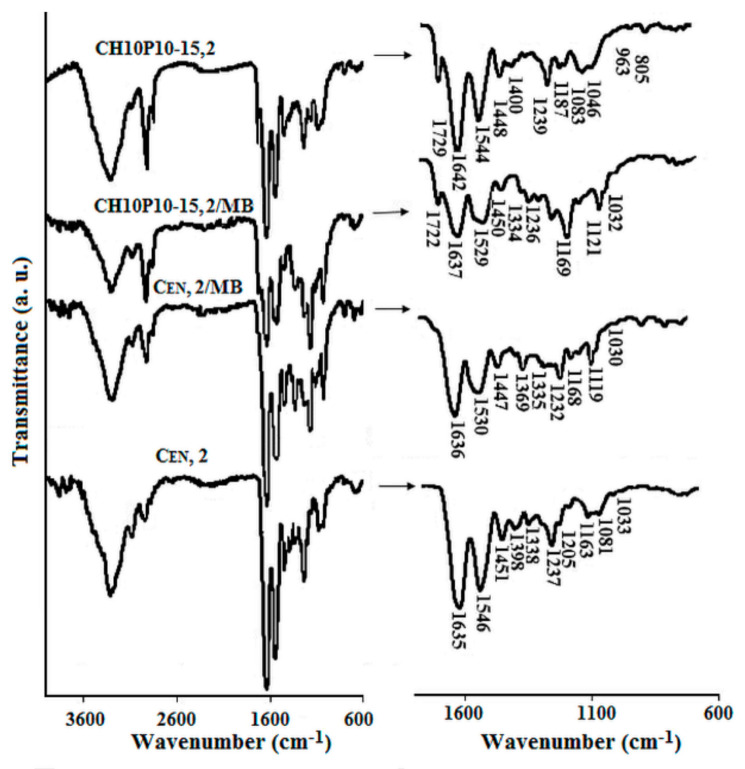
Representative FTIR spectra of the investigated samples before and after MB loading.

**Figure 6:**
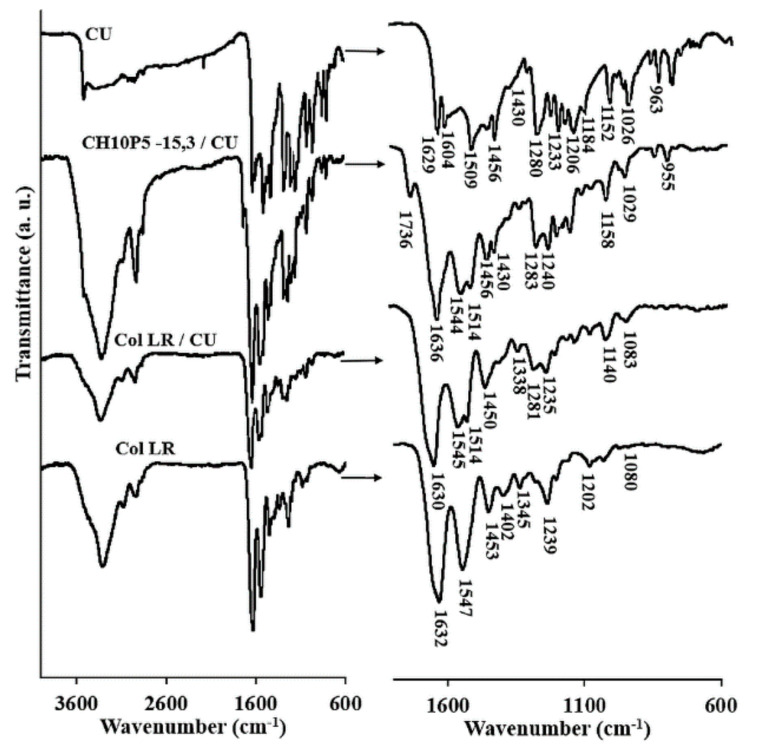
Comparative FTIR spectra of the CU loaded samples.

To compare the in vitro drug delivery of collagen-based samples of various structures, these were immersed in the solutions of two different drug models—methylene blue (hydrophilic) and curcumin (hydrophobic, with extremely low solubility in aqueous medium). The FT-IR spectra (Figure 5 and Figure 6) confirmed the drugs’ loading, showing several absorbance bands from both hydrogels and drugs. The typical four amide bands for collagen [32] situated at 3313 cm^−1^ (ν NH- amide A), 3077 cm^−1^ (ν NH- amide B), 1637 cm^−1^ (ν C=O -amide I) and 1546 cm^−1^ (δ NH coupled with the ν C–N absorption- amide II), are maintained in the loaded specimens, with slight modifications. For instance, the spectra of the samples loaded with MB show a splitting of the amide A band, together with an enlargement and shift to lower wavenumber values (1529 cm^−1^) of the amide II band, indicating a specific interaction between the protein and the drug model. The superposition with the C=C, C=N, C–C and C–N bonds’ stretching vibrations in the MB heterocycle [33] is also worth considering. The intensification of the absorption band at about 2928 cm^−1^ could be associated with the contribution of the CH_3_ stretching vibrations of the dimethylamino groups. The domain from 1420 cm^−1^ to 1300 cm^−1^, characteristic for δ CH_3_ and δ CH_2_ wagging in collagen, is modified after MB loading. The band situated at 1339 cm^−1^, specific to δ CH in protein, is moved to 1336 cm^−1^, and increased by superposition with the ν_het_ C=S and ν C–N in N–CH_3_ vibrations in MB. New signals appeared, which can be also attributed to MB [33], at 1171 cm^−1^ (δ_het_ C–H), at about 1120 cm^−1^ (δ_het_ C–N), 1031 cm^−1^ and 1007 cm^−1^ (γ_het_ C–H), 702 cm^−1^ (ν C–N + γ C–S–C) + δ C–H, δ C–S–C, δ_het_ C–C).

Modifications (band intensity increase, band shifting, and the appearance of new bands) related to the presence of the drug and its interaction with the polymer support can be also observed in the spectra of the curcumin-loaded samples (Figure 6). For the collagen-GAG-PCL structures, even the ν OH sharp band specific to curcumin can be seen, but it has shifted from 3518 cm^−1^ to 3505 cm^−1^, most probably due to this group’s involvement in intermolecular hydrogen bonds. Again, the band at about 2924 cm^−1^, assigned to ν C–H, is strongly increased. The amide band I is shifted downwards to about 1629 cm^−1^ due to superposition with ν C=O and ν C=C absorptions of the aromatic ring in curcumin. The main new bands, ascertained to curcumin presence [34], appear at 1510 cm^−1^ (mixed vibrations: ν C=O, δ C–C, δ C–C=O), 1428 cm^−1^ (δ C–H), 1205 cm^−1^ (δ C=C–H), 1185 cm^−1^ (δ C–O–C), 1158 cm^−1^ (δ C–O–C), 1030 cm^−1^ (ν C–H) and 962 cm^−1^ (ν C–O). The band at 1279 cm^−1^, attributed to mixed deformation vibrations in the keto form of curcumin (δ C=C–C, δ C=C–H, δ C–OH), is increased. For the Col LR sample, the bands attributed to δ C–O–C and ν C–O, in particular, are shifted to lower wavenumber values, i.e., to 1162 cm^−1^, 1135 cm^−1^ and 956 cm^−1^. This aspect may be related to the strong interaction of curcumin with collagen, as this nutraceutical is known to act as a cross-linker [35]. For the hybrid structures, the interaction of curcumin with the polyester (hydrophobic type, hydrogen bonding), the lower amount of the protein in the formulation, and its involvement in packed complex generation with GAG, must be considered.

The evaluation of drug models loading by spectrophotometry gave the results presented in Table 2 and Table 3.

It can be observed that both MB and CU loading are less influenced by the samples’ porosity, but are highly dependent on their formulation. The MB-loaded amount decreases with the increasing of the cross-linking degree, and increases with the –COOH group’s content (i.e., resulting from polyester scission under UV irradiation, for longer durations [26]), as evidenced for samples CP_30_-20,2 and C_EN_P_2_-30,2. However, the presence of hyaluronic acid derivative did not improve the MB loading, as expected, considering the specific electrostatic interactions between them. This is most probably due to competitive interpolymer GAG-protein interactions, with the MB-DMSHA interactions being weakened.

The included curcumin amount increases with the PCL content in the matrix formulation, supporting the influence of hydrophobic interactions.

**Figure 7:**
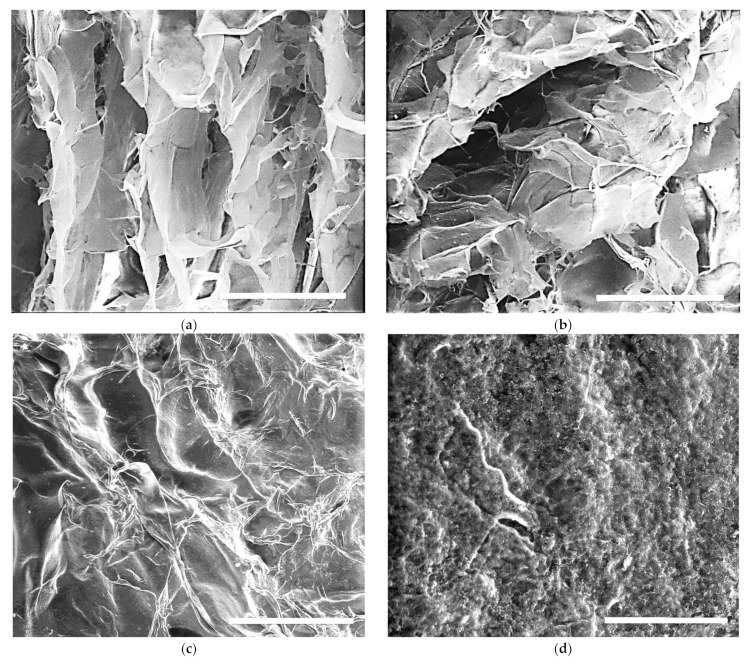

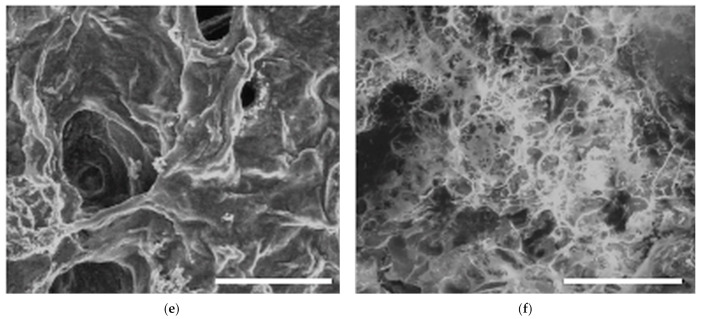
SEM microphotographs for different collagen–based sponges before and after curcumin loading: (**a**) Col LR; (**b**,**c**) curcumin-loaded Col LR; (**d**,**e**) CH_10_P_10_-15,2, surface and cross section images; (**f**) CU loaded CH_10_P_10_-15,2. Scale bar: (**a**–**c**) −400 μm; (**d**) −100 μm; (**e**,**f**) −200 μm.

The SEM observation revealed modifications of morphological features by drug loading, i.e., an increased disorder, and smaller pores (Figure 7). In the case of curcumin-loaded samples, the walls in Col LR structure seem to be thicker, while, for the hybrid porous materials, it is evident that the deposition of curcumin occurs mainly at the walls’ surface, even inside the pores.

#### 3.2.2. Drug Delivery

**Figure 8:**
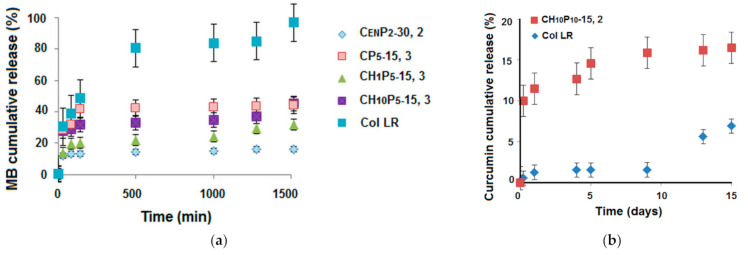
Cumulative release profile for representative MB-loaded (**a**) and CU-loaded (**b**) collagen-based sponges.

Drug release is an important parameter that affects bioavailability. The drug release plots (Figure 8) suggest, in this case, a combination of diffusion and degradation-controlled delivery. Sponges of similar porosity were evaluated. As can be observed, the interaction of MB with DMSHA in the hybrid matrices, or with -COOH groups in C_EN_P_2_-30,2, resulted in a sustained drug delivery after an initial rapid release (in the first 80 min). The release rate can be tailored by composition control.

For the control collagen sample loaded with CU, the degradation-controlled release is the main delivery mechanism. The delivery is mainly observed beginning with day 15, most payloads being released by structure defibration after 20 days. A similar behaviour also occurred for the sample C_EN_P_2_-30,2, with greater release occurring after 21 days (due to a higher cross-linking degree compared to Col LR). Sample CH_10_P_10_-15,2 gave rise to a more efficient, sustained delivery, due to the contribution of both the diffusion of absorbed CU and of the degradation-controlled release of CU implied in cross-linking. Degradation of the DMSHA component takes place first [18], and hyaluronic acid is known to have a protecting/solubilisation effect on CU. From the kinetics data, it can be supposed that it might be suitable to use hyaluronic acid for hybrid matrices in order to enhance the solubility and bioavailability of CU. On the other hand, it is worth noting that HA conjugated with anti-cancer drugs is considered to work as a “smart bullet” in anti-cancer therapy [36], while collagen films that incorporated curcumin were demonstrated to be an efficient approach for dermal wound healing [37].

The results of the antibacterial activity against *S. aureus* of the curcumin-loaded samples confirmed the evidenced delivery aspects. After 24 h, the Col LR reference sample without curcumin degraded, while the curcumin-loaded sample gave rise to a partial inhibition zone (11 mm), maintaining its integrity. Sample CH_10_ P_10_-15,2 loaded with curcumin exhibited an inhibition zone of 21 mm.

## 4. Conclusions

Hybrid collagen-based 3D structures comprising PCL and a hyaluronic acid derivative (in dense, porous or macroporous form) were investigated in comparison with a commercially available collagen sponge, for intended applications in the biomedical area (for wound dressing or in lab models). According the obtained data, all samples favour water absorption and moisture vapour penetration, but only the porous/macroporous samples exhibited a comparable behaviour with the commercial product demonstrating the important effect of porosity, followed by the crosslinking degree and formulation, on such properties. The hybrid formulation, a proven strategy for improving pharmacokinetics, gave rise to a sustained drug release for both model drugs used in this study—MB and CU. The upload and the delivery kinetics were mainly dependent on formulation (specific drug- matrix components interactions) and on cross-linking degree. The release profile was found to be diffusion- and erosion-controlled, each contribution being formulation dependent. Curcumin, acting as a cross-linker for collagen, delayed the release from the Col LR control sample, in accordance with the delayed degradation, with a burst phase release at the collagen matrix disruption point.

As a consequence, the best results were obtained for a content of 10% DMSHA (5% HA) and 10% PCL, due to a sequential degradation and the ability of degradation products of GAG to facilitate CU dissolution in aqueous media. The antibacterial effect increases positively with CU concentration; thus, this sample had the best antibacterial behaviour against *S. aureus*, achieving an appropriate equilibrium between diffusion and erosion through its composition, and thereby allowing the delivery of an appropriate amount of curcumin.

The recommended properties (water uptake and water vapour sorption) for such hybrid collagen-based sponges are formulations with 5–10% DMSHA and 5–10% PCL, as an optimal range in the studied series, for possible application as a wound dressing or in lab models. These may also especially improve curcumin loading and delivery to wound sites.

## Figures and Tables

**Table 1:** Investigated collagen-based matrices. Composition and certain characteristics.

Code	ESR (g × g^−1^)	Porosity (%)
Col LR	27.9 ± 0.3	93.5 ± 0.5
CH_1_P_5_-15,1	5.2 ± 0.2	67.0 ± 1.0
CH_10_P_5_-15,1	3.7 ± 0.1	59.0 ±1.5
CH_10_P_30_-15,1	2.8 ± 0.1	54.0 ± 0.5
CP_5_-10,2	38.5 ± 0.4	94.0 ± 0.9
CP_5_-20,2	30.1 ± 0.3	94.0 ± 0.6
CP_15_-10,2	27.5 ± 0.2	93.9 ± 0.5
CP_15_-20,2	26.0 ± 0.5	94.5 ± 0.6
CP_30_-10,2	19.5 ± 0.5	91.2 ± 0.9
CP_30_-20,2	17.5 ± 0.7	92.0 ± 1.1
C_EN_,2	19.0 ± 0.8	95.6 ± 0.5
C_EN_P_2_,2	15.0 ± 0.3	92.8 ± 1.0
C_EN_P_2_-30,2	28.0 ± 0.5	94.9 ± 1.4
CP_5_-15,3	27.0 ± 0.3	94.4 ± 0.5
CH_1_P_5_-15,3	15.0 ± 0.2	94.0 ± 0.4
CH_10_P_5_-15,3	11.0 ± 0.1	93.0 ± 0.3
CH_10_P_10_-15,2	7.0 ± 0.1	70.0 ± 0.2
CH_10_P_50_-15,3	6.0 ± 0.2	57.0 ± 0.8

**Table 2:** Spectrophotometrical data on MB loading in collagen-based samples.

Code	Loading Capacity (mg MB/g Sample)
Col LR	28.5 ± 1.0
CH_1_P_5_-15, 1	23.8 ± 1.5
CH_10_P_5_-15, 1	14.4 ± 1.8
CP_5_-10, 2	25.4 ± 2.0
CP_5_-20, 2	17.6 ± 1.6
CP_15_-10,2	13.1 ± 1.2
CP_15_-20,2	18.7 ± 1.0
CP_30_-10,2	22.3 ± 1.7
CP_30_-20,2	37.9 ± 1.0
C_EN_,2	25.9 ± 0.8
C_EN_P_2_,2	32.2 ± 1.0
C_EN_P_2_-30,2	36.1 ± 0.5
CH_10_P_10_-15,2	14.0 ± 0.5
CP_5_-15, 3	27.9 ± 1.0
CH_1_P_5_-15, 3	18.0 ± 0.7
CH_10_P_5_-15, 3	14.9 ± 0.3
CH_10_P_50_-15, 3	13.0 ± 0.6

**Table 3:** Curcumin loading capacity of the investigated samples.

Code	Loading Capacity (%)
Col LR	12.7 ± 0.4
C_EN_P_2_-30,2	14.7 ± 0.1
CH_10_P_5_-15,3	15.0 ± 0.4
CH_10_P_10_,2	15.4 ± 0.3
CH_10_P_30_-15,1	16.5 ± 0.2
CH_10_P_50_-15,3	17.2 ± 0.5

## Data Availability

Data supporting the findings of this study are contained within the article and are available from the corresponding author upon request.
